# Irrational use of antibiotics without a clinical diagnosis: a short case report

**DOI:** 10.4314/ahs.v23i2.24

**Published:** 2023-06

**Authors:** Rajat Agarwal, Mohd Mubeen, Harpreet Singh

**Affiliations:** 1 All India Institute of Medical Sciences Deoghar, India; 2 Max Super Speciality Hospital, Shalimar Bagh, Delhi, India

**Keywords:** Antibiotics, infective endocarditis, fever, infection

## Abstract

Antibiotics are often started irrationally and continued injudiciously worldwide without a clinical diagnosis. Antibiotic prescription practises are governed by multiple factors like lack of diagnostic facilities, secondary infections, poor sanitary conditions, easy availability, pharmaceutical companies' marketing strategies and patients seeking the fastest cure from healthcare providers. We report a case of a 40-year-old male who was treated intermittently with multiple antibiotics for 3 months without being clinically diagnosed. The patient continued having episodes of fever with brief periods of remission. The occurrence of recurrent episodes of transient ischemic attacks alarmed him, and he was brought to a tertiary hospital, where a diagnosis of culture-negative Infective Endocarditis (IE) was made. He underwent successful high-risk double valve replacement and was discharged from the hospital in satisfactory condition.

**Key messages:**

Antibiotics should be started judiciously with a proper clinical indication and should be reviewed from time to time regarding selection, duration, and response. In the case of non-responders, a thorough clinical examination followed by relevant investigations should be done for a proper clinical diagnosis.

## Introduction

Injudicious and prolonged use of antibiotics (>28 days) 1 without a clinical diagnosis is a common practise worldwide. There are multiple factors like absence of diagnostic facilities, patient pressure for quick recovery, self-medication, lack of awareness, easy availability of drugs etc. responsible for it.^2^ This has led to antimicrobial resistance (AMR), a major future problem predicted by World Health Organisation (WHO).^3^ Even the programmes developed by WHO to combat antimicrobial resistance require a dedicated protocol for their execution.^3^ We report a case of 40-year-old male treated with an empirical/span>intermittent course of multiple antibiotics for a prolonged duration of 3 months without a clinical diagnosis, until he was admitted to a tertiary hospital.

## Case history

A 40-year-old man presented to a tertiary hospital with chief complaints of high-grade fever for 3 months and breathlessness for 1 month. He had a sudden onset of fever 3 months back and consulted a local physician. The fever was continuous and high grade. He was treated with intermittent courses of multiple antibiotics (Table 1) and other supportive treatment. His spikes of fever persisted with weekly relapses. In the last month, his dyspnoea has gotten worse. During this period, he was also diagnosed with diabetes mellitus. He also had a history of recurrent episodes of transient ischemic attack (TIA) for 1 month. He got repeated complete blood counts and chest X-rays for the persistent fever. His past medical history (beyond 3 months) was unremarkable.

**Table 1:** Antibiotics administered to the patient

S.no.	Antibiotic	Route	Dosage	Frequency	Duration(days)
1.	Cefuroxime	Oral	500mg	BD	5
2.	Piperacillin-Tazobactam	IV	4.5g	TDS	7
3.	Linezolid	Oral	600mg	BD	5
4.	Meropenem	IV	500mg	TDS	7
5.	Amikacin	IV	500mg	BD	5
6.	Augmentin	Oral	625mg	TDS	7
7.	Clarithromycin	Oral	500mg	BD	5
8.	Ceftriaxone	IV	2g	BD	7
9.	Vancomycin	IV	1g	BD	21
10	Meropenem	IV	1g	TDS	14

S.no. 1-7: Before admission to the hospital.

S.no. 8-9: In the hospital before surgery

S no. 9-10: During surgery and for 14 days afterwards.

IV: intravenous, mg: milligram, g: gram, BD: 2 times a day, TDS: 3 times a day.

His clinical examination at admission revealed a fever above 100-degree Fahrenheit, sinus tachycardia (pulse rate of 134/minute), a respiratory rate of 28/minute and decreased bilateral breath sounds. His oxygen saturation at room temperature was 92%. His laboratory tests at admission showed low haemoglobin (8g/dl), high white blood cell count (17.6 x 10^3^ cells/mm^3^), elevated C-reactive protein (25mg/L), raised serum creatinine (1.7 mg/dl), deranged liver function tests (Serum bilirubin-2.2 mg/dl, alanine aminotransferase-78 U/L, aspartate aminotransferase-76 U/L) and glycosylated haemoglobin of 6.9%. Paired blood and sputum cultures turned out to be negative. An X-ray of the chest revealed bilateral (B/L) pleural effusion (PE). Contrast enhanced computed tomography (CECT) of the chest and abdomen was suggestive of B/L PE, mild pericardial effusion, ground glass opacities in both lower lobes of the lungs, splenomegaly, and a bulky right kidney. A diagnostic pleural tap was negative for tuberculosis. Urine culture revealed significant growth of Candida species.

A differential diagnosis of pneumonia, right pyelonephritis, and fungal urinary tract infection was made. The patient was started on Ceftriaxone (2g IV BD), Vancomycin (1g IV BD), Fluconazole (200 mg IV BD) and other supportive treatment.

In view of persistent spikes of fever and worsening dyspnoea echocardiography was done after 2 days. It revealed IE with large vegetations on the mitral (2.1x0.9 cm) and aortic (0.9x0.9 cm) valves with severe mitral regurgitation and aortic regurgitation with normal left ventricular (LV) FUNCTION and moderate right ventricular (RV) DYSFUNCTION.

**Figure 1:**
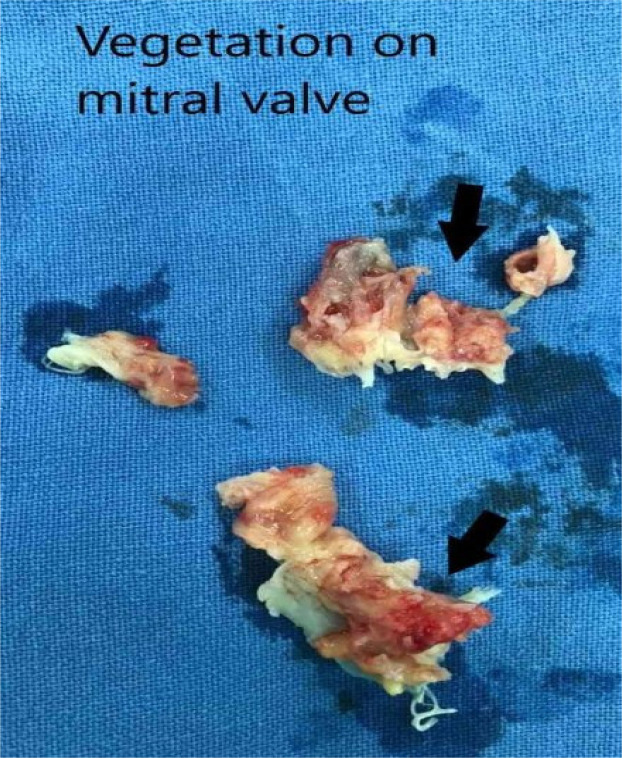
Large vegetation. (Without arrow). Destroyed leaflets of mitral valve as shown by arrows

The case was referred to the cardiothoracic surgery team for surgical opinion. The team recommended urgent surgery due to heart failure, intermittent fever, and large vegetations to avoid embolization. The case was a high-risk surgery in view of RV DYSFUNCTION, large mobile vegetations, deranged liver function tests, and a history of recurrent episodes of TIA. But in this case, the patient will never recover unless those large vegetations are removed, and in the absence of surgery, there will be a very high risk of embolism of vegetations to the brain and other organs. Finally, after informed consent with mention of high risk from the patient and his family, double valve replacement via median sternotomy under cardiopulmonary bypass was done. Both leaflets of the mitral valve were destroyed by huge vegetation (Figure 1).

**Figure 2:**
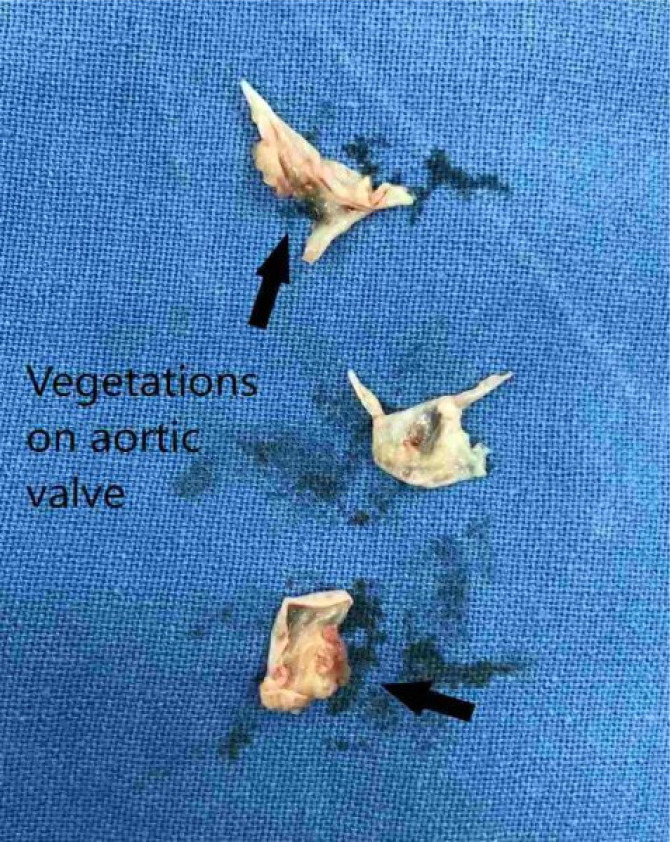
Large vegetations on aortic valve as shown by arrows. Perforation in aortic leaflet (without arrow)

Similarly, there were multiple vegetations on the aortic valve with perforations in the non-coronary cusp (Figure 2). Postoperatively, patients developed low cardiac output, which was managed by vasopressors. There were episodes of supraventricular arrhythmias which were managed by amiodarone. There was a development of moderate PE managed by diuretics. After the above stormy postoperative course, the patient recovered well and was discharged satisfactorily. The culture reports (bacterial and fungal) of the excised aortic and mitral valves, sent during the operation, turned out to be sterile. The patient was given the necessary antibiotics (Table1) for the recommended duration.

## Discussion

Irrational use and starting of antibiotics without a clinical diagnosis is very common practice. This often masks the true disease and further delays the diagnosis. 5 According to one study, most antibiotics used for persistent fever in four low- and middle-income countries are Watch antibiotics, a class of antibiotics thought to have a high resistance potential.^6^

In the above-mentioned case, the patient was continued on empiric antibiotics with persistent fever and no clinical suspicion or diagnosis was attempted. The clinical diagnosis might have led to the usage of antibiotics according to culture and sensitivity, and this in turn could have avoided the formation of large vegetations. Appropriate duration of antibiotics is a must for any successful treatment and to avoid the development of antibiotic resistance. It was only when the patient developed a transient ischemic attack that he was alarmed by the deteriorating situation. Any patient with persistent fever should have a thorough history, physical examination, blood and radiological investigations performed at the appropriate time. IE is a deadly disease with a high in-hospital mortality of 31.3%.^7^

The prior administration of antibiotics is the single most important cause of culture-negative endocarditis and leads to prolonged and noxious management.^8^ The outcome of IE depends on a high index of clinical suspicion, proper antimicrobial treatment, and urgent surgical intervention when indicated. 9 The presence of large vegetations in the above patient, combined with symptoms of heart failure, were major indicators of urgent surgery. The vegetations were classified as bacterial in this case because they were large and had destroyed the leaflets, as opposed to nonbacterial vegetations, which are small and surround the margins of valve leaflets.^10^

Bacterial vegetations lead to recurrent fever in contrast to nonbacterial vegetations, which are often incidentally diagnosed. 10 The irrational and overuse of expensive antibiotics can also pose an economic burden on patients. Thus, a proper awareness needs to be created for the judicious use of antibiotics with optimal duration and proper indications for optimal treatment to avoid the biggest concern of present times, antibiotic resistance. Every time someone prescribes antibiotics, the indication, choice, response, duration, and compliance should be carefully considered in order to achieve the best possible results.
